# Cognitive Functions Among Pupils in Schools Near and Around an Electronic Waste Recycling Site at Agbogbloshie in Accra, Ghana

**DOI:** 10.3390/toxics13080615

**Published:** 2025-07-23

**Authors:** Serwaa A. Bawua, Kwame M. Agbeko, Ibrahim Issah, Afua A. Amoabeng-Nti, Saskia Waldschmidt, Katja Löhndorf, Thomas Küpper, Jonathan Hogarh, Julius N. Fobil

**Affiliations:** 1 Department of Biological, Environmental & Occupational Health Sciences, School of Public Health, University of Ghana, Accra P.O. Box LG13, Ghana; makwagbeko123@gmail.com (K.M.A.); iissah@ug.edu.gh (I.I.); aamoabeng@ug.edu.gh (A.A.A.-N.); 2 West Africa Center for Global Environmental & Occupational Health, College of Health Sciences, University of Ghana, Accra GA-270-0053, Ghana; 3 Institute of Occupational and Social Medicine, Aachen Technical University, 52074 Aachen, Germany; swaldschmidt@ukaachen.de (S.W.); tkuepper@ukaachen.de (T.K.); 4 Faculty for Travel Medicine, Royal College of Physicians and Surgeons of Scotland, Glasgow 232-242, UK; 5 Department of Environmental Science, Kwame Nkrumah University of Science & Technology, Kumasi GPS AK-448-4944, Ghana; jhogarh@gmail.com

**Keywords:** Agbogbloshie, cognitive functions, e-waste, Intelligence Quotient (IQ), neurotoxicity, Wechsler Intelligence Scale for Children (WISC)

## Abstract

**Background:** Electronic waste (e-waste) recycling in informal settings like Agbogbloshie in Accra, Ghana, releases toxic metals into the environment, posing serious health risks to nearby residents, particularly children. This study assessed the body burdens of lead (Pb), manganese (Mn), cadmium (Cd), chromium (Cr), nickel (Ni), and arsenic (As) and their association with cognitive function in schoolchildren living within 1 km of the Agbogbloshie site. **Method:** A cross-sectional study involving 56 pupils collected demographic data and blood and urine samples and administered the Wechsler Intelligence Scale for Children—Fourth Edition (WISC-IV). Blood was tested for Pb and Mn and urine for Cd, Cr, Ni, and As. Associations between metal levels and cognitive outcomes were examined using regression analyses, adjusting for confounders. **Result:** Children showed elevated metal levels, with mean blood Pb of 60.43 µg/L and urinary s of 21.50 µg/L. Symptoms of cognitive dysfunction were common: 75% reported confusion, 67.9% poor memory, and 66% poor concentration. Urinary Cr levels were significantly associated with lower Full-Scale IQ (β = −18.42, *p* < 0.05) and increased difficulty in decision-making (OR = 0.1, *p* < 0.05). **Conclusion:** These findings underscore the neurodevelopmental risks of heavy metal exposure from e-waste in low- and middle-income countries and call for urgent public health interventions and policy actions.

## 1. Introduction

Informal e-waste recycling, which is common in Low- and Middle-Income Countries (LMICs), involves hazardous practices such as open burning, dismantling, and acid leaching, releasing toxic metals and organic pollutants into the environment [1]. These contaminants pose severe health risks, particularly to vulnerable populations, including children, who are more susceptible to the neurotoxic effects of heavy metals due to their underdeveloped nervous systems [2,3].

Agbogbloshie, located in Accra, Ghana, is one of the largest informal e-waste recycling sites in Africa and has been identified as a global pollution hotspot [4]. Workers and nearby residents, including schoolchildren, are routinely exposed to harmful substances such as lead (Pb), manganese (Mn), cadmium (Cd), chromium (Cr), nickel (Ni), and arsenic (As) [5,6]. Exposure to these metals has been linked to a range of adverse health outcomes, including neurodevelopmental deficits [7,8]. Studies have shown that even low levels of heavy metal exposure can significantly impair cognitive function, affecting memory, attention, learning, and overall intellectual ability in children [9]. Given the growing concerns regarding environmental contamination from e-waste processing, understanding its potential neurodevelopmental effects is critical.

Despite the well-documented risks associated with heavy metal exposure [10], limited research has investigated the impact of environmental metal exposure on cognitive function among schoolchildren living near e-waste recycling sites in Sub-Saharan Africa. While studies have examined the effects of Pb and Mn exposure on cognitive function in children from high-income countries [11], there is a lack of region-specific data on the relationship between exposure to multiple metals, including Cr, Ni, and As, and neurodevelopment in children from e-waste-impacted environments. Furthermore, most existing studies have focused on occupational exposure among adult e-waste workers, with minimal attention given to the unintended exposure of children through contaminated air, water, soil, and food [12].

Filling this gap is crucial, as children living near e-waste recycling sites may experience chronic, low-dose exposure that could negatively affect their cognitive development, academic performance, and overall well-being. Generating data on this population will inform public health interventions, policy regulations, and community-based mitigation strategies to reduce heavy metal exposure and its long-term consequences.

This study aims to (1) evaluate the body burden of Pb and Mn (in blood) and Cd, Cr, Ni, and As (in urine) among schoolchildren attending schools near the Agbogbloshie e-waste recycling site; and (2) assess the association between metal concentrations and cognitive function using standardized neuropsychological assessments, including the Wechsler Intelligence Scale for Children—Fourth Edition (WISC-IV). Findings from this study will contribute to the growing evidence on the neurodevelopmental impacts of environmental pollution and provide critical insights for mitigating associated risks due to exposure among vulnerable children. In addition, the results will support policy recommendations aimed at improving e-waste management practices and protecting children from hazardous environmental exposure.

## 2. Materials and Methods

### 2.1. Study Design

This study utilized a cross-sectional design to assess the association between environmental metal exposure and cognitive function among schoolchildren. The study involved a combination of questionnaire-based surveys, biological sample collection, laboratory analysis, and cognitive assessments.

### 2.2. Study Area

**Figure 1:**
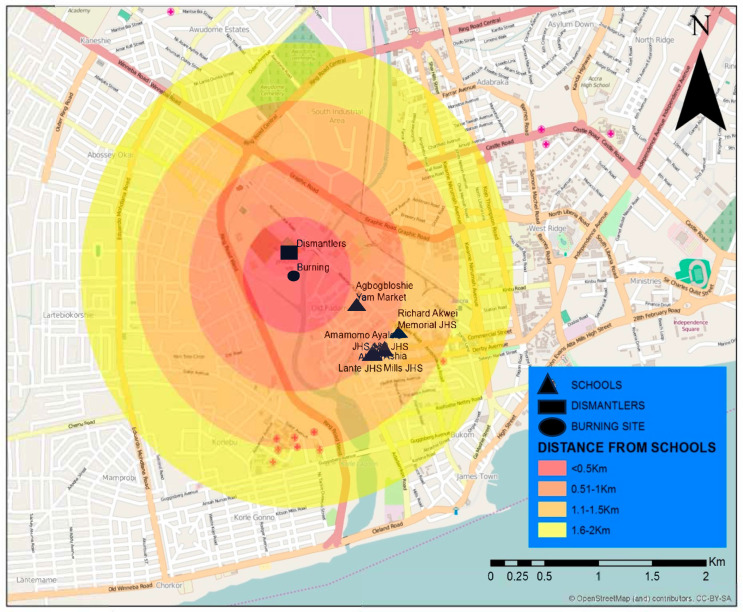
Map showing the Agbogbloshie e-waste site and study schools. Source: adapted from OpenStreetMap and contributors, CC-BY-SA available on Google Map.

The study was conducted in four schools located near the Agbogbloshie e-waste recycling site in Accra, Ghana. Agbogbloshie is one of the largest informal e-waste recycling hubs in Africa, where electronic waste is dismantled, burned, and processed under hazardous conditions. The site spans approximately 15 acres and is situated west of the Odaw River. Nearby communities are densely populated and predominantly house informal workers engaged in e-waste recycling activities. The study schools, Ayalolo 1&2 Junior High School (JHS), Amamomo JHS, Ashia Mills JHS, and Richard Akwei Memorial School, are located within a 1 km radius of the recycling site (Figure 1). These schools were selected based on their proximity to Agbogbloshie and their high student populations, which allowed for adequate sampling. The area is characterized by poor air quality, high levels of soil contamination, and significant environmental health risks [6].

### 2.3. Participant Recruitment

Participants were recruited from the four selected schools using a randomized sampling approach. Eligibility criteria included (i) current enrolment in one of the study schools, (ii) willingness to follow the study protocol, (iii) written informed consent from a biological parent or guardian and assent from the pupil, and (iv) the absence of diagnosed mental health disorders. School authorities and the Metropolitan Directorate of the Ghana Education Service were consulted and provided written consent for the study. An information session was held to explain the study’s purpose, risks, and benefits to pupils and their guardians. A total of 56 pupils were randomly selected and invited to participate. Those whose parents agreed voluntarily provided blood and urine samples for laboratory analysis. Ethical approval for the study was granted by the University of Ghana Review Board at the Noguchi Memorial Institute for Medical Research (NMIMR-IRB CPN 058/14-15), ensuring compliance with research guidelines.

### 2.4. Data Collection

#### 2.4.1. Questionnaire Survey

A structured questionnaire was administered to assess demographic characteristics, environmental exposure history, and cognitive function symptoms among pupils. The questionnaire was adapted from validated toxicity and neurodevelopmental assessment tools and included sections on socio-demographic factors (age, sex, years of schooling, household socioeconomic status), medical history (history of neurological disorders, head trauma, or chronic illnesses), and environmental exposure (proximity to e-waste activities, parental occupation, and household exposure sources). The cognitive function assessment component included self-reported symptoms such as memory difficulties, confusion, poor concentration, learning disabilities, and decision-making challenges. To enhance accuracy, the questionnaire was carefully presented to the students using simple, age-appropriate language. Prior to data collection, the questionnaires were reviewed with teachers to ensure clarity and relevance. During administration, trained field staff conducted face-to-face interviews with the pupils and provided additional clarification as needed. Teachers were present throughout the process to support comprehension and to help maintain a focused and comfortable environment for students to respond honestly. Furthermore, the students’ responses, especially regarding cognitive symptoms, were verified through observation and inputs from teachers, and, where necessary, follow-up verification with parents. These measures were taken to reduce subjectivity and strengthen the validity of the findings.

#### 2.4.2. Blood and Urine Sample Collection

Blood and urine samples were collected following standardized biosafety and quality assurance protocols. A trained phlebotomist and physician collected venous blood samples (3 mL) using a sterile butterfly syringe into metal-free EDTA-coated tubes. Urine samples (50 mL) were collected in acid-washed, pre-labeled polyethylene containers after the pupils washed their hands with soap and water. Midstream urine collection was emphasized to minimize contamination. All samples were immediately stored on ice at −20 °C before being transported to the University of Ghana for temporary storage. The samples were then shipped to the Institute of Occupational and Social Medicine at RWTH Aachen University, Germany, for analysis. Chain-of-custody protocols were strictly followed to maintain sample integrity.

#### 2.4.3. Laboratory Analysis

Blood and urine samples were analyzed using High-Resolution Continuum Source Atomic Absorption Spectrophotometry (H-RCS AAS) ContrAA700 (Analytik Jena, Jena, Germany), following standard protocols recommended by the Deutsche Forschungsgemeinschaft (DFG) and the European Chemical Industry Council [13,14]. Briefly, blood samples were diluted in matrix modifier reagents and analyzed for Pb and Mn. Urine samples were acidified with 65% nitric acid (HNO_3_), vortexed, heated to 50–60 °C, and analyzed for Cd, Cr, Ni, and As. Detection limits were 10 μg/L (Pb), 5 μg/L (Mn), and 0.2 μg/L (Cd, Cr, Ni, As). Deionized water was used for sample dilution and container cleaning.

#### 2.4.4. Cognitive Function Analysis

Cognitive function was assessed using a modified toxicity questionnaire to evaluate symptoms such as poor memory, confusion, poor concentration, poor coordination, difficulty making decisions, stuttering/stammering, slurred speech, learning disabilities, and inattentiveness. Additionally, IQ scores were measured using the Wechsler Intelligence Scale for Children—Fourth Edition (WISC-IV) [15]. This standardized neuropsychological assessment provided objective measures of cognitive ability and potential neurotoxic effects associated with heavy metal exposure.

### 2.5. Statistical Analysis

Descriptive statistics were used to summarize participant characteristics, with continuous variables presented as means and standard deviations and categorical variables as frequencies and percentages. Associations between metal concentrations and Full-Scale IQ were analyzed using multivariable linear regression models, adjusting for age and parental smoking status. Multivariable logistic regression models were used to examine associations between heavy metal concentrations and cognitive dysfunction symptoms, with results reported as odds ratios (ORs) with 95% confidence intervals (CIs). A significance level of *p* < 0.05 was considered statistically significant. We also employed Pearson correlation matrices to assess linear and monotonic associations between individual metal concentrations. All analyses were performed using STATA version 13 (Stata Corp., College Station, TX, USA).

## 3. Results

### 3.1. Socio-Demographic Characteristics of Pupils

The majority of the participants were female (55.4%), with a mean age of 14.6 (±1.20) years. The average body weight was 52.1 (±16.8) kg, and the mean height was 158.6 (±8.2) cm. Approximately 62.5% of the pupils had attended their current school for 1 to 6 years. Regarding maternal education, 85.8% of the pupils’ mothers had completed middle school or junior high school. The marital status of parents indicated that 60.7% of pupils had married parents, while 17.9% reported divorced parents, and 10.7% had single or widowed parents. Concerning financial resources, 41.1% of pupils had at least 3 cedis available for daily school expenses. The highest proportion of pupils attended Ashia Mills JHS (36%), followed by Amamomo JHS (22%), Ayalolo 1&2 JHS (21%), and Richard Akwei JHS (21%) (Table 1).

### 3.2. Prevalence of Cognitive Dysfunction Symptoms

The prevalence of cognitive dysfunction symptoms among pupils is presented in Table 2. The most frequently reported conditions were confusion (75.0%), poor memory (67.9%), and poor concentration (66.0%). Additional symptoms included poor coordination (26.8%), difficulty making decisions (35.8%), stuttering/stammering (12.5%), slurred speech (19.6%), learning disabilities (33.9%), and inattentiveness (37.5%). These findings suggest a high burden of neurocognitive impairments among pupils attending schools near the Agbogbloshie e-waste site.

### 3.3. Metal Concentrations in Blood and Urine

The mean concentrations of metals in blood and urine samples are presented in Table 3. Blood lead (Pb) had the highest concentration at 60.43 (±45.7) µg/L, followed by urine arsenic (As) at 21.50 (±43.9) µg/L. Blood manganese (Mn) was recorded at 5.51 (±6.5) µg/L, while urine nickel (Ni) was 5.32 (±4.1) µg/L. The lowest detected concentrations were urine cadmium (Cd) at 0.13 (±0.3) µg/L and urine chromium (Cr) at 0.15 (±0.3) µg/L (Table 3).

### 3.4. Association Between Metals and Full-Scale IQ

Table 4 presents the relationship between heavy metal concentrations and Full-Scale IQ scores. The overall model was not significant, F (6, 93) = 1.52, *p* = 0.18, with an R^2^ = 0.07. However, urinary Cr levels showed a statistically significant negative association with IQ (β = −18.42, 95% CI [2.01, 34.84], *p* < 0.05), indicating that elevated Cr exposure may impair cognitive development. Other metals, including blood Pb, blood Mn, urine Cd, urine Ni, and urine As, did not demonstrate significant associations with IQ in either crude or adjusted models.

### 3.5. Associations Between Metal Exposure and Cognitive Functions

We used multivariable logistic regression models to examine the association between metal concentrations and cognitive impairments (Table 5). The results of the analysis showed that urine Cr was significantly associated with difficulty making decisions (OR = 0.1, 95% CI [1.77 × 10^−8^–0.19], *p* = 0.018), implying that increased urinary Cr levels are linked to a higher likelihood of decision-making difficulties. We did not observe any significant associations between the other metals and cognitive dysfunction symptoms (Table 5).

### 3.6. Correlation Heatmap of Metal Concentrations

**Figure 2:**
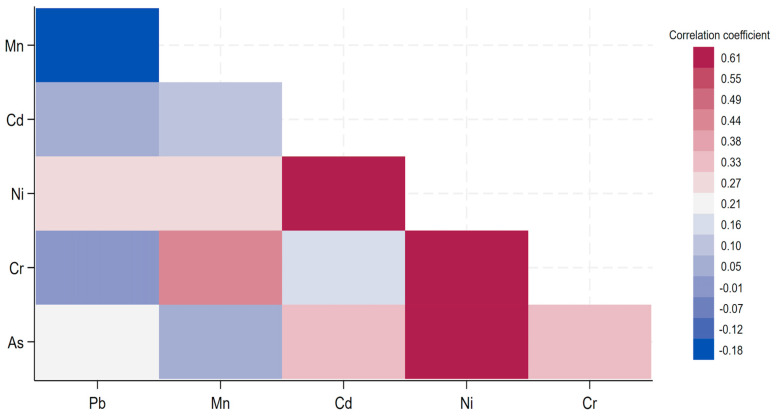
Correlation heatmap of metal concentrations.

The Pearson correlation heatmap of metal concentrations revealed a moderate to low correlation among each pair of metal concentrations, as the correlation coefficients were all below 0.70 (Figure 2)

## 4. Discussion

This study examined the body burden of toxic metals (blood Pb and Mn and urinary Cd, Cr, Ni, and As) and their association with cognitive and neurological functions in school children exposed to e-waste recycling activities. The findings showed high levels of blood Pb and urinary As, while levels of blood Mn and urinary Cd, Cr, and Ni were lower. Multivariable linear regression analyses revealed a significant association between the mean concentrations of urinary Cr and Full-Scale IQ after adjusting for age. Similarly, using a multivariable logistic regression analysis, a significant association was observed between urinary Cr levels and difficulty making decisions.

The findings of this study differ from several prior studies that have linked heavy metal exposure to cognitive deficits in children. For example, Wasserman et al. (2006) found that elevated Mn levels in drinking water negatively affected cognitive abilities in Bangladeshi children [16], while Bouchard et al. (2007) reported associations between Mn exposure and behavioral issues in children [17]. In a review by Liu et al. (2020), a relationship between blood Mn levels and decreased IQ scores was identified [18]. Similarly, Pb exposure has been extensively associated with cognitive decline and behavioral impairments in children [19]. In contrast, our study did not find significant associations between Mn or Pb levels and cognitive outcomes, suggesting possible differences in exposure pathways, individual susceptibility, or methodological factors.

The significant association between urinary Cr levels and difficulty making decisions is a novel finding, as previous research has not widely reported similar associations with Cr exposure. For instance, a recent systematic review and meta-analysis by Islam et al. (2022) did not show any significant difference in Cr exposure between children with cognitive deficits and healthy control children [20]. Chromium, particularly in its hexavalent form (Cr VI), is primarily known for its carcinogenic and respiratory effects, but emerging evidence suggests that it may contribute to oxidative stress and neurotoxicity, potentially affecting cognitive processes [21].

Several plausible biological mechanisms have been proposed to explain chromium’s neurotoxic effects [22,23]. Cr VI readily crosses cell membranes via anion transporters and is reduced intracellularly to Cr III, generating reactive oxygen species (ROS) in the process [24]. This redox cycling induces oxidative stress, disrupts mitochondrial function, damages DNA, and triggers apoptosis in neuronal cells [25,26]. Moreover, chromium-induced inflammation in the central nervous system can alter synaptic signaling, impair neurogenesis, and interfere with neurotransmitter pathways critical to learning, memory, and executive functioning [23,27]. These mechanisms support the observed association between urinary Cr levels and deficits in IQ and decision-making in this study and require further mechanistic investigation in a larger population. The lack of significant associations with other metals may reflect the influence of confounding factors, such as the dietary intake of competing nutrients [28], interactions among co-exposed metals with antagonistic or synergistic properties [29], and interindividual genetic variation in metal metabolism and detoxification [30].

To explore potential co-exposure effects, we generated a Pearson correlation heatmap (Figure 2) of the measured metal concentrations. This analysis revealed generally low to moderate correlations between metals, with all pairwise correlation coefficients falling below 0.70. This suggests limited collinearity among the metals and provides some insight into their independent distribution within this population. Although we recognize the analytical complexity of mixture methods (e.g., weighted quantile sum regression, Bayesian kernel machine regression), future studies should incorporate mixture modeling approaches to better capture potential interactive or synergistic effects of multiple metals on neurodevelopment.

A key strength of this study is its focus on a vulnerable population, school children living near an e-waste recycling site, where data on metal exposure and cognitive outcomes are limited. In addition, metals were measured objectively using blood and urine samples and employed standardized cognitive assessments to evaluate neurodevelopmental impacts. However, some limitations must be considered. First, the cross-sectional study design precludes causal inference and limits our ability to assess the long-term impacts of chronic exposure. While biomarkers such as blood Pb may reflect recent exposure (e.g., past 1–3 months), they may also capture elements of longer-term exposure through equilibrium with bone stores. Still, this does not substitute for the prospective tracking of exposure–outcome relationships over time. Second, potential confounders such as nutritional status, socioeconomic factors, and prenatal exposures were not accounted for in detail. These limitations may have contributed to the lack of significant associations between most metals and cognitive function. Lastly, regarding the multiple comparisons presented in Table 5, we acknowledge that the possibility of chance findings cannot be ruled out; hence, the interpretation of the significant associations should therefore be approached with caution.

The findings highlight the urgent need for further research with more robust methodological designs that can inform public health interventions to reduce heavy metal exposure within e-waste recycling communities. Although this study did not find widespread associations between metals and cognitive impairment, the elevated Pb levels remain a concern given their well-documented neurotoxic effects. Implementing safer waste management policies, increasing community awareness, and establishing routine biomonitoring programs are crucial steps to mitigate exposure risks. Additionally, clinical and nutritional interventions such as chelation therapy for children with significantly elevated blood lead levels and proactive vitamin and mineral supplementation (e.g., iron, calcium, vitamin C) should be explored as strategies to minimize metal absorption and toxicity [31,32].

Furthermore, the role of cognitive assessments such as IQ testing in identifying at-risk children should be emphasized beyond diagnostic purposes. IQ results can guide individualized educational planning, behavioral support, and academic interventions tailored to children’s needs. Establishing school-based screening programs in high-risk communities could facilitate early detection and referral for supportive services.

Future research should adopt longitudinal study designs to assess the long-term effects of heavy metal exposure on neurodevelopment. In addition, studies should explore genetic and nutritional factors that may modify susceptibility to heavy metal toxicity. Expanding research to include larger, more diverse populations and investigating the potential synergistic and/or antagonistic effects of multiple metal exposures will provide a clearer understanding of the risks associated with e-waste recycling environments while also identifying actionable solutions to mitigate those risks.

## 5. Conclusions

This study provides valuable insights into the association between heavy metal exposure and cognitive function among school children living near an e-waste recycling site. While no significant relationships were observed between Pb, Mn, Cd, As, or Ni concentrations and cognitive function, an association was found between urinary Cr levels and difficulty making decisions. This finding underscores the need for further investigation into the neurodevelopmental effects of Cr exposure. In addition, the high levels of Pb detected in the study population highlight the ongoing risk posed by e-waste contamination.

## Figures and Tables

**Table 1:** Socio-demographic characteristics of pupils in the study schools.

Characteristics	Frequency (*n* = 56)	Percentage (%)	Mean (±SD)
Gender of respondents			
Male	25	44.6	
Female	31	55.4	
Age (years)			14.6 (1.20)
Weight (kg)			52.1 (16.8)
Height (cm)			158.6 (8.2)
Years in present school compound			
1 year	19	33.9	
2 years	3	5.4	
3 years	3	5.4	
5 years	4	7.1	
6 years	6	10.7	
7 years	13	23.2	
8 years	7	12.5	
10 years	1	1.8	
Mother’s highest education			
Never schooled	17	30.4	
Primary school	14	25	
Middle school/junior high school	17	30.4	
Senior high school	5	8.9	
Institute/tertiary	1	1.8	
Marital status of parents			
Married	34	60.7	
Divorced	10	17.9	
Separated	6	10.7	
Widowed	6	10.7	
Amount spent at school in a day			
1 cedi	12	21.4	
2 cedis	21	37.5	
3 cedis and above	23	41.1	
School attended			
Ayalolo 1&2 JHS	12	21	
Ashia Mills JHS	20	36	
Amamomo JHS	12	22	
Richard AM JHS	12	21	

SD = standard deviation; JHS = junior high school; AM = Akwei Memorial.

**Table 2:** Prevalence of cognitive dysfunction symptoms among pupils (*n* = 56).

Cognitive Function	Never (%)	Occasionally (Not Severe) (%)	Occasionally (Severe) (%)	Frequently (Not Severe) (%)	Frequently (Severe) (%)	Total Prevalence (%)
Poor Memory	32.1	46.4	10.7	9.0	1.8	67.9
Confusion	25.0	62.5	10.7	1.8	0.0	75.0
Poor Concentration	40.0	57.1	5.3	3.6	0.0	66.0
Poor Coordination	73.2	19.6	3.6	1.8	1.8	26.8
Difficulty Making Decisions	64.2	25.0	3.6	3.6	3.6	35.8
Stuttering/Stammering	87.5	5.3	3.6	1.8	1.8	12.5
Slurred Speech	80.4	3.6	7.1	5.3	3.6	19.6
Learning Disability	66.1	16.1	7.1	10.7	0.0	33.9
Inattentiveness	62.5	23.2	3.6	7.1	3.6	37.5

**Table 3:** Metal concentrations in blood and urine (*n* = 56).

Metal (μg/L)	Exposure Medium	Mean Concentration (μg/L)	Standard Deviation (±SD)
Pb	Blood	60.43	45.7
Mn	Blood	5.51	6.5
Cd	Urine	0.13	0.3
Cr	Urine	0.15	0.3
Ni	Urine	5.32	4.1
As	Urine	21.50	43.9

**Table 4:** Association between heavy metal concentrations and full-scale IQ (*n* = 56).

Metal	β	95% CI	*p*-Value
B-Pb	0.01	(−0.06, 0.09)	0.783
B-Mn	−0.48	(−1.16, 0.21)	0.166
U-Cd	−5.12	(−21.01, 10.77)	0.520
U-Cr	−18.42	(2.01, 34.84)	0.029 *
U-Ni	−0.38	(−0.82, 1.57)	0.529
U-As	−0.01	(−0.11, −0.08)	0.770
Adjusted coefficient of determination is 3%			

Note: we quantified the effect of each metal on Full-Scale IQ, controlling for the confounding effect of the age of the pupil and parental smoking status; β = regression coefficient; * *p* < 0.05 indicates statistical significance.

**Table 5:** Association between metals and cognitive impairments (*n* = 56).

	Poor Memory		Confusion		Poor Concentration		Poor Coordination		Difficulty Making Decisions	
**Metal**	**OR (95% CI)**	***p***	**OR (95% CI)**	***p***	**OR (95% CI)**	***p***	**OR (95% CI)**	***p***	**OR (95% CI)**	***p***
B-Pb	1.01 (0.98, 1.02)	0.446	1.02 (0.99–1.06)	0.200	1.01 (0.98–1.02)	0.414	1.01 (0.99–1.02)	0.478	1.02 (0.98–1.05)	0.223
B-Mn	1.01 (0.88, 1.15)	0.917	1.01 (0.88–1.17)	0.847	1.02 (0.89–1.16)	0.798	1.08 (0.93–1.25)	0.334	1.14 (0.98–1.32)	0.090
U-Cd	10.14 (0.14, 71.2)	0.287	1.78 (0.03–118.46)	0.787	1.49 (0.06–39.34)	0.809	0.69 (0.00–166.45)	0.898	47.07 (0.56–3967.91)	0.089
U-Cr	2.95 (0.10, 85.64)	0.530	1.26 (0.04–35.60)	0.893	5.54 (0.13–234.28)	0.370	0.00 (5.49–2.13)	0.084	0.00 (1.77 × 10^−8^–0.19)	0.018
U-As	0.98 (0.95, 1.00)	0.103	0.99 (0.97–1.02)	0.675	0.99 (0.96–1.01)	0.160	1.02 (0.98–1.05)	0.239	1.00 (0.98–1.03)	0.843
U-Ni	0.89 (0.71, 1.12)	0.341	0.94 (0.73–1.21)	0.613	0.91 (0.73–1.13)	0.401	0.84 (0.59–1.18)	0.309	1.13 (0.89–1.43)	0.328
	Stuttering/Stammering		Slurred Speech		Learning Disability		Inattentiveness			
Metal	OR (95% CI)	*p*-value	OR (95% CI)	*p*-value	OR (95% CI)	*p*-value	OR (95% CI	*p*-value		
B-Pb	0.99 (0.97–1.03)	0.872	0.99 (0.98–1.02)	0.978	1.02 (0.99–1.04)	0.202	0.99 (0.97–1.01)	0.285		
B-Mn	1.04 (0.84–1.29)	0.719	0.93 (0.77–1.13)	0.468	1.01 (0.88–1.15)	0.926	0.96 (0.83–1.11)	0.568		
U-Cd	4.69 (0.12–190.49)	0.413	1.42 (0.04–46.18)	0.842	5.55 (0.22–137.48)	0.295	21.04 (0.25–1801.43)	0.180		
U-Cr	1.58 (0.02–154.49)	0.845	1.19 (0.02–93.45)	0.938	0.49 (0.01–17.75)	0.701	0.54 (0.01–23.32)	0.751		
U-As	0.99 (0.97–1.02)	0.592	1.00 (0.98–1.03)	0.655	0.99 (0.97–1.01)	0.384	0.98 (0.94–1.01)	0.201		
U-Ni	1.02 (0.72–1.44)	0.919	1.04 (0.81–1.32)	0.770	1.02 (0.82–1.27)	0.843	1.18 (0.92–1.52)	0.195		
Abbreviations: OR—odds ratio; 95% CI—95% confidence interval. Models were adjusted for age and maternal smoking.										

Note: we quantified the effect of each metal on cognitive impairments, controlling for the confounding effect of the age of the pupil and parental smoking status.

## Data Availability

The original contributions presented in the study are included in the article; further inquiries can be directed to the corresponding author.
